# Reproducibility of ultrasound-derived fat fraction in measuring hepatic steatosis

**DOI:** 10.1186/s13244-024-01834-1

**Published:** 2024-10-22

**Authors:** Danlei Song, Pingping Wang, Jiahao Han, Huihui Chen, Ruixia Gao, Ling Li, Jia Li

**Affiliations:** 1 Department of Ultrasound, Zhongda Hospital, Medical School, Southeast University, Nanjing, China; 2 Department of Endocrinology, Zhongda Hospital, Medical School, Southeast University, Nanjing, China

**Keywords:** Steatotic liver disease, Ultrasound, Ultrasound-derived fat fraction, Reproducibility

## Abstract

**Purpose:**

Steatotic liver disease (SLD) has become the most common cause of chronic liver disease. Nevertheless, the non-invasive quantitative diagnosis of steatosis is still lacking in clinical practice. This study aimed to evaluate the reproducibility of the new parameter for steatosis quantification named ultrasound-derived fat fraction (UDFF).

**Materials and methods:**

The UDFF values were independently executed by two operators in two periods. In the process, repeated measurements of the same patient were performed by the same operator under different conditions (liver segments, respiration, positions, and dietary). Finally, the results of some subjects (28) were compared with the MRI-derived proton density fat fraction (PDFF). The concordance analysis was mainly achieved by the intraclass correlation coefficient (ICC) and Bland–Altman.

**Results:**

One hundred-five participants were included in the study. UDFF had good reliability in measuring the adult liver (ICC_intra-observer_ = 0.96, ICC_inter-observer_ = 0.94). Meanwhile, the ICC of the two operators increased over time. The variable measurement states did not influence the UDFF values on the surface, but they affected the coefficient of variation (Cov) of the results. Segment 8 (S8), end-expiratory, supine, and fasting images had the most minor variability. On the other hand, the UDFF value of S8 displayed satisfied consistency with PDFF (mean difference, −0.24 ± 1.44), and the results of both S5 (mean difference: −0.56 ± 3.95) and S8 (mean difference: 0.73 ± 1.87) agreed well with the whole-liver PDFF.

**Conclusion:**

UDFF measurements had good reproducibility. Furthermore, the state of S8, end-expiration, supine, and fasting might be the more stable measurement approach.

**Critical relevance statement:**

UDFF is the quantitative ultrasound parameter of hepatic steatosis and has good reproducibility. It can show more robust performance under specific measurement conditions (S8, end-expiratory, supine, and fasting).

**Trial registration:**

The research protocol was registered at the Chinese Clinical Trial Registry on October 9, 2023 (http://www.chictr.org.cn/). The registration number is ChiCTR 2300076457.

**Key Points:**

- There is a lack of non-invasive quantitative measurement options for hepatic steatosis.
- UDFF demonstrated excellent reproducibility in measuring hepatic steatosis.
- S8, end-expiratory, supine, and fasting may be the more stable measuring condition.
- Training could improve the operators’ measurement stability.
- Variable measurement state affects the repeatability of the UDFF values (Cov).

**Graphical Abstract:**

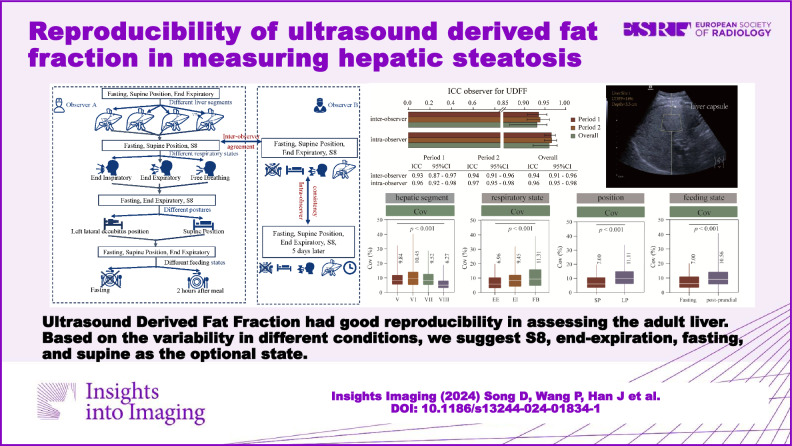

## Introduction

Steatotic liver disease (SLD) is a comprehensive term encompassing all causes of hepatic steatosis, mainly including metabolic dysfunction-associated steatotic liver disease (MASLD), alcohol-related liver disease (ALD), and MASLD with increased alcohol intake [1]. Hepatocellular steatosis is the essential clinical characteristic of SLD [2]. ALD has demonstrated a relatively stable prevalence in recent years, but the obesity pandemic has driven a sustained increase in the global incidence of MASLD to approximately 38% [3, 4], making it the most common chronic liver disease worldwide. MASLD has also emerged as the fastest-growing cause of chronic liver failure, hepatocellular carcinoma, and liver transplantation [5]. The current gold standard for diagnosing SLD remains liver biopsy, but this method is invasive and carries the potential risks of bleeding and infection [6]. Therefore, exploring effective noninvasive diagnostic methods for hepatic steatosis is necessary.

Non-invasive imaging techniques are becoming essential in the early screening, diagnosis, and outcome assessment of hepatic steatosis. MRI-derived proton density fat fraction (PDFF) is the standard imaging biomarker for diagnosing fatty liver [7]. However, MRI cannot be used for routine evaluation due to its time-consuming and complex operation. US has the advantages of safety, being radiation-free, non-invasive, and inexpensive, and is recommended as the preferred diagnostic procedure for SLD. However, conventional 2D ultrasound is limited by subjectivity and operator dependency [8]. In recent years, new parameters related to the quantitative diagnosis of hepatic steatosis have been rapidly developed, such as attenuation coefficient (AC), backscatter coefficient (BSC), and signal-to-noise ratio. However, the diagnostic accuracy, performance reliability, and scientific robustness of these imaging parameters are still under clinical investigation, which limits their widespread use in clinical diagnosis [9]. The newly developed parameter named ultrasound-derived fat fraction (UDFF) can effectively detect and quantify hepatic steatosis. The calculation of this parameter combines AC (detection of energy loss per unit distance in the tissue) and BSC (detection of differential scattering per unit volume per unit steradian angle), which quantitatively measures both the liver echo enhancement and the posterior beam attenuation [10]. UDFF is quantified as the percentage of liver steatosis, therefore, it can be directly compared with MRI-PDFF to provide similar clinical applications [11]. Certainly, UDFF is more convenient and cost-effective than PDFF. Meanwhile, it is a quantitative parameter that is not as susceptible to operator influence as conventional US. In addition, most of the currently available techniques for quantitative assessment of hepatic steatosis are based on AC, whereas UDFF incorporates the calculation of BSC, which takes into account the changes in the microstructure of the tissue while measuring energy loss. Therefore, UDFF may suit well as the diagnostic tool for hepatic steatosis. Furthermore, the imaging system equipped with UDFF can estimate hepatic fibrosis by the auto-pSWE parameter. Compared to pSWE, auto-pSWE provides 15 pSWE values in seconds, significantly reducing imaging time.

The main objective of this study was to assess the reproducibility of UDFF measurements and explore the contributing factors. Further, the optimal measurement state of UDFF was initially investigated by measuring under different conditions and using the MRI-PDFF as the reference standard.

## Materials and methods

### Subject

The studies involving human participants were reviewed and approved by the IEC for Clinical Research of Zhongda Hospital, Affiliated with Southeast University [no. 2023ZDSYLL116-P01]. All participants provided written informed consent before the ultrasound examination. We have registered the protocol in the Chinese Clinical Trial Registry (ChiCTR2300076457). This study was a single-center cross-sectional study. We prospectively recruited patients who visited the Endocrinology Department and underwent liver scans due to the risk of fatty liver (diabetes, abnormal liver enzymes overweight, etc.) from October to December 2023. Inclusion conditions were subjects at risk of hepatic steatosis who could complete breath-holding and fasting tests (age ranges from 18 to 75 years), and who were willing to participate in this study and provide informed consent forms. Patients with severe underlying diseases, pregnant, children, patients with thoracic deformities whose ultrasound detectable area is smaller than the required measurement area, and patients who cannot cooperate were excluded. Based on the above inclusion and exclusion criteria, we ultimately included 105 participants. Clinical data of the subjects were collected, including age, sex, height, weight, waist circumference, hip circumference, distance from the skin to the liver capsule (SCD), and appropriate laboratory tests.

### Sample size

The sample size of the study was calculated by PASS Software (version 23.0). Assuming the inter-observer ICC for UDFF or auto-pSWE measurements can reach 0.7 and at least not less than 0.5. A minimum sample size of 87 patients was calculated with a 90% power and 5% significance.

### UDFF and auto-pSWE measurements

All ultrasound examinations were completed on the Acuson Sequoia ultrasound scanner (Siemens Healthiness). The investigation period was conducted in two consecutive periods (period 1 and period 2) of 1 month and 2 months, respectively. The timing of the staged experiments was designed to assess the effect of training on reliability. Two observers independently performed the liver measurements. Observer A had three years of ultrasonography experience and Observer B had one year’s experience. However, neither operator had prior measurement experience with UDFF and auto-pSWE. Participants fasted for at least 4 h before being examined. The patient abducts the right arm to its maximum extent during measurements to fully expose the intercostal space. Liver morphology was first assessed using Grayscale ultrasonography by the curvilinear probe (5C1, 1.0–5.7 MHz). Subsequently, UDFF and auto-pSWE measurements were conducted separately by the deep abdominal transducer (DAX, 1.0–3.5 MHz). The manufacturer pre-defined the fixed and unchangeable ROI depth and size (1.5 cm from the liver capsule; 3 cm × 3 cm). The operator should align the positioning horizontal line and liver capsule to ensure accurate measurement depth. Firstly, the measurement consistency of UDFF and auto-pSWE was evaluated. The steps were as follows: both Observers A and B measured the UDFF and auto-pSWE values of each subject under the same state to assess inter-observer agreement, and Observer B repeated the UDFF and auto-pSWE measurement on the same patient under the same measurement state after 5 days to assess intra-observer agreement. A valid measurement of UDFF is considered the successful display of a value on the report page. Repeated the UDFF measurements five times under each state and took the median as the final value. Meanwhile, the number of attempts to derive the five valid values under each measurement state should not exceed ten, otherwise, this measurement state will be recorded as a missing value. A valid measurement for auto-pSWE is defined as having at least ten sub-ROIs with numerical displays, IQR/Med (SWV) ≤ 0.3 and IQR/Med (E) ≤ 0.6. Four auto-pSWE measurements were taken at the same location for each patient and their median was taken. The number of attempts to complete the four valid values under each measurement state should not exceed 8, otherwise, this measurement state will be recorded as a missing value. Further, operator A repeated UDFF measurements at different hepatic segments, respiration, feeding, and postural states to explore the factors influencing UDFF. The variable of hepatic segments included segments V, VI, VII, and VIII; the variable of respiratory status included end of inspiratory (EI), end of expiratory (EE), and free breathing (FB); the variable of feeding status included fasting for 4 h and post-feeding for 2 h; the variable of postural status included lateral position (LP) and supine position (SP). The measurement efficiency of each state is defined as the ratio of successfully measured individuals to the total number.

### Qualitative and semi-quantitative analysis of hepatic steatosis

The visual grading and hepatic renal index (HRI) are qualitative and semi-quantitative analyses of hepatic steatosis, respectively [12]. These two parameters in this study were assessed by the experienced ultrasound doctor who was not aware of the other results (Observer C). The diagnostic basis of visual grading is as follows [13]. Grade 0: the echogenicity of liver parenchyma is slightly greater than or equal to the renal cortex, with visible periportal and diaphragmatic echogenicity. Grade 1: increased hepatic echogenicity, with visible periportal and diaphragmatic echogenicity; Grade 2: increased hepatic echogenicity with impaired visualization of periportal echogenicity, without obscuration of the diaphragm. Grade 3: increased hepatic echogenicity with impaired visualization of periportal echogenicity and obscuration of the diaphragm. The HRI was the echo intensities between the liver parenchyma and the right renal cortex. The ROI sizes set in the liver parenchyma and kidney parenchyma were 150 mm^2^ and 50 mm^2^, respectively. The ROI was placed near the image’s central region to maintain consistent depth, gain, and pixel intensity. Image analysis was conducted using the Image J software. HRI measurements were repeated three times for each liver and mean values were calculated.

### MRI PDFF technique

Measurements were taken in some participants without MRI contraindications (metal implants, etc.). The liver MRI examination (Siemens Healthiness) was performed within the week following the US. The participants took the mDixon-Quant examination in the SP and cephalo-caudal direction. MRI-PDFF was obtained using a 6-echo acquisition during a single breath hold. The technical parameters of the sequence were as follows: imaging plane, axial; TR, 9.00 ms, TE, 1.05–7.38 ms, field of view, 280 × 320 mm; acquisition matrix, 111 × 160; reconstruction matrix, 640 × 640; flip angle, 4°; section thickness, 3.5 mm; acquisition voxel, 2.8 × 2.8 × 3.5 mm; and reconstruction voxel, 1.4 × 2.4 × 3.5 mm. The liver was segmented into eight segments following the Couinaud segmentation method. The PDFF estimation was obtained by placing three elliptic ROIs of 200 ± 20 mm² in each hepatic segment. The mean values were taken for further analysis. The overall average of the PDFF in eight liver segments was considered the whole liver’s measurement value. ROIs were manually set in the homogeneous liver parenchyma 5 mm away from the capsule, avoiding focal lesions, biliary tract, gallbladder, and large vessels.

### Statistical analysis

Statistical analyses were performed using SPSS software (version 26, IBM). The intra- and inter-observer reproducibility in measuring ultrasound parameters was evaluated by the intraclass correlation coefficient (ICC). The ICC values were interpreted according to the proposed standards: < 0.50 poor, 0.50–0.75 moderate, 0.75–0.90 good, and > 0.90 excellent. The differences in UDFF value under various conditions were analyzed by the Kruskal–Wallis test (the data did not satisfy a normal distribution). The coefficient of variation (Cov) is the ratio of the within-subject standard deviation to the within-subject mean, and the smaller the value, the better the reproducibility of the data. Cov was further used to assess the reproducibility of the UDFF values. The correlation between UDFF and the visual grade of hepatic steatosis was evaluated with the Spearman rank correlation coefficient, and the relevance between UDFF and HRI was analyzed with Pearson’s correlation coefficient. Factors affecting UDFF and auto-pSWE values were analyzed by univariate and multivariate linear regression. Draw the Bland–Altman plot to assess the difference between UDFF and PDFF measurements. All the statistical tests used a two-sided test, with a *p* value less than 0.05 as the statistically significant difference test.

## Results

### Baseline characteristics

**Fig. 1:**
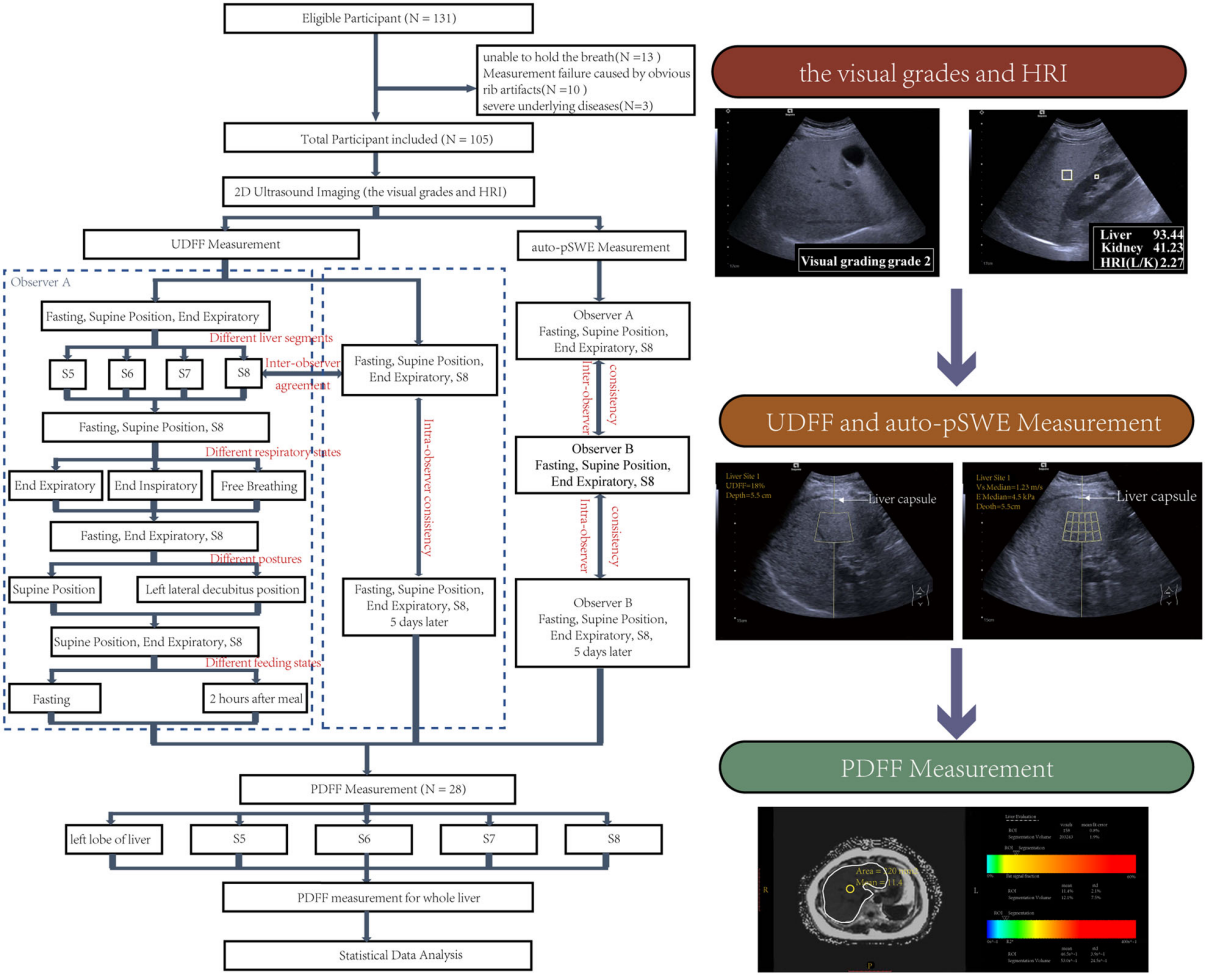
Study flowchart. In total, 105 participants were included in this study. Finally, 28 of 105 subjects (26%) underwent the measurement of MRI-PDFF. The illustration gives the US and MRI procedures in a patient with moderate steatosis

Of the 131 patients who underwent US examination during the study period, 13 patients were unable to hold their breath, the measurement of 10 patients failed due to obvious rib artifacts, and 3 patients had serious underlying diseases (Fig. 1). Thus, a total of 105 adult volunteers were finally included in this study (69 males and 36 females, mean age 52 years, age range 18–75 years). Participants were divided into the non-SLD Group (33 participants with a grade 0) and the SLD Group (72 participants with a visual grade greater than 0) based on the visual grade of hepatic steatosis. Participants’ demographic information and all measured variables are listed in Tables 1, 2, and 3. Some of the measurement states of the UDFF were not adequately measured during the study. The efficiency rate of the measurements in S5, S6, and S7 and end-inspiration was 98.10%, 84.76%, 93.33%, and 99.05%, respectively.

**Table 1:** Baseline characteristics of the 105 participants

	Non-SLD, (*N* = 33)	SLD, (*N* = 72)	*p* value
Sex, (female)	11 (33.33%)	25 (34.72%)	0.890
Sex, (male)	22 (66.67%)	47 (65.27%)	
Age, (yr)	61.00 ± 17.25	51.50 ± 25.00	0.032
Height, (m)	167.10 ± 7.80	169.0 ± 8.50	0.239
Weight, (kg)	64.10 ± 10.60	76.6 ± 14.60	< 0.0001
BMI	22.90 ± 3.10	26.6 ± 3.60	< 0.0001
Waistline, (cm)	83.80 ± 9.80	93.2 ± 9.70	< 0.0001
Hipline, (cm)	91.90 ± 5.70	98.5 ± 7.40	< 0.0001
SCD, (cm)	1.90 ± 0.40	2.30 ± 0.50	< 0.0001
whr	0.90 ± 0.10	0.90 ± 0.10	0.059
whtr	0.50 ± 0.10	0.60 ± 0.10	0.0001
HRI	1.46 ± 0.75	1.02 ± 1.06	0.003
VFA	55.75 ± 42.25	89.37 ± 47.5	< 0.0001
diabetes	29 (87.88%)	69 (95.83%)	0.539
ALT, (U/L)	19.61 ± 9.00	30.87 ± 26.50	0.010
AST, (U/L)	17.71 ± 5.00	23.17 ± 11.50	0.017
γ-GGT, (U/L)	17.58 ± 9.50	37.04 ± 26.75	< 0.0001
TBIL, (μmol/L)	9.65 ± 4.35	11.15 ± 6.12	0.110

*SCD* skin–liver capsule distance, *whr* waist–hip ratio, *whtr* waist–height ratio, *VFA* visceral fat area

**Table 2:** The UDFF of 105 participants in different measurement states

UDFF, (%)	Non-SLD, (*N* = 33)	SLD, (*N* = 72)	*p* value	The efficiency rate, (%)
Fasting, SP, EE (V)	4.52 ± 1.52 (*N* = 33)	13.90 ± 8.05 (*N* = 70)	< 0.0001	98.10
Fasting, SP, EE (VI)	3.96 ± 1.43 (*N* = 24)	12.69 ± 9.25 (*N* = 65)	< 0.0001	84.76
Fasting, SP, EE (VII)	4.64 ± 1.91 (*N* = 28)	14.83 ± 8.01 (*N* = 70)	< 0.0001	93.33
Fasting, SP, EE, VIII	5.06 ± 2.03 (*N* = 33)	15.40 ± 8.94 (*N* = 72)	< 0.0001	100.0
Fasting, SP, VIII (EI)	4.84 ± 1.95 (*N* = 32)	15.24 ± 8.39 (*N* = 72)	< 0.0001	99.05
Fasting, SP, VIII (FB)	4.97 ± 2.30 (*N* = 33)	15.39 ± 8.35 (*N* = 72)	< 0.0001	100.0
Fasting, EE, VIII (LP)	4.73 ± 2.35 (*N* = 33)	13.83 ± 7.84 (*N* = 72)	< 0.0001	100.0
SP, EE, VIII (feeding)	5.00 ± 2.08 (*N* = 33)	14.99 ± 8.23 (*N* = 72)	< 0.0001	100.0
Fasting, SP, EE, VIII (another radiologist)	5.15 ± 2.39 (*N* = 33)	14.96 ± 8.74 (*N* = 72)	< 0.0001	100.0
Fasting, SP, EE, VIII (5 days later)	4.97 ± 1.94 (*N* = 33)	14.78 ± 8.47 (*N* = 33)	< 0.0001	100.0

*EI* end of inspiration, *FB* free breathing, *LP* lateral position

**Table 3:** The auto-pSWE values of 105 participants

Auto-pSWE, (m/s; kPa)	Non-SLD, (*N* = 33)	SLD, (*N* = 72)	*p* value	The efficiency rate, (%)
Mean SWV, (m/s)	1.16 ± 0.22	1.10 ± 0.19	0.09	100.0%
Mean SWV, (m/s) (another radiologist)	1.14 ± 0.21	1.10 ± 0.23	0.14	100.0%
Mean SWV, (m/s) (5 days later)	1.13 ± 0.27	1.08 ± 0.29	0.16	100.0%

### Inter-observer and intra-observer variability of the values

**Fig. 2:**
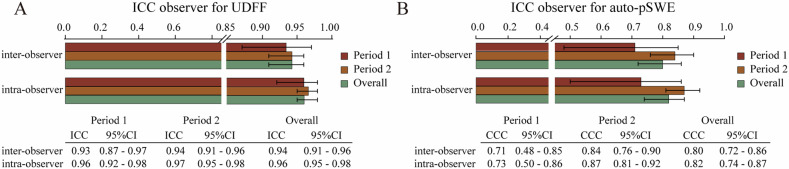
The inter- and intra-observer ICCs and 95% CIs of measurement for overall and each period. **A** The measurements of UDFF. **B** The measurements of auto-pSWE

The ICC for intra-observer repeatability and inter-observer of UDFF was 0.96 (95% CI: 0.95–0.98) and 0.94 (95% CI: 0.91–0.96), respectively (Fig. 2A). And the intra-observer and inter-observer ICC of auto-pSWE were 0.82 (95% CI: 0.74–0.87) and 0.80 (95% CI: 0.72–0.86), respectively (Fig. 2B). The ICCs of measurements for UDFF and auto-pSWE are all greater than 0.75, indicating that they have good repeatability. For the UDFF measurement, the intra-observer ICCs in period 1 and period 2 were 0.96 and 0.97, and the inter-observer ICCs in the two periods were 0.93 and 0.94, respectively, whereas for auto-pSWE measurement, the intra-observer ICCs in the two periods were 0.73 and 0.87, and the inter-observer ICCs in period 1 and period 2 were 0.71 and 0.84, respectively. The result of the two periods showed that the ICCs of both techniques are higher in period 2 than in period 1, which indicates that training improves the stability of the measurements. The ICCs of UDFF were greater than that of auto-pSWE in different periods, which indicates that the measurement repeatability of UDFF is better than that of auto-pSWE.

### Differences in UDFF values under different conditions

**Fig. 3:**
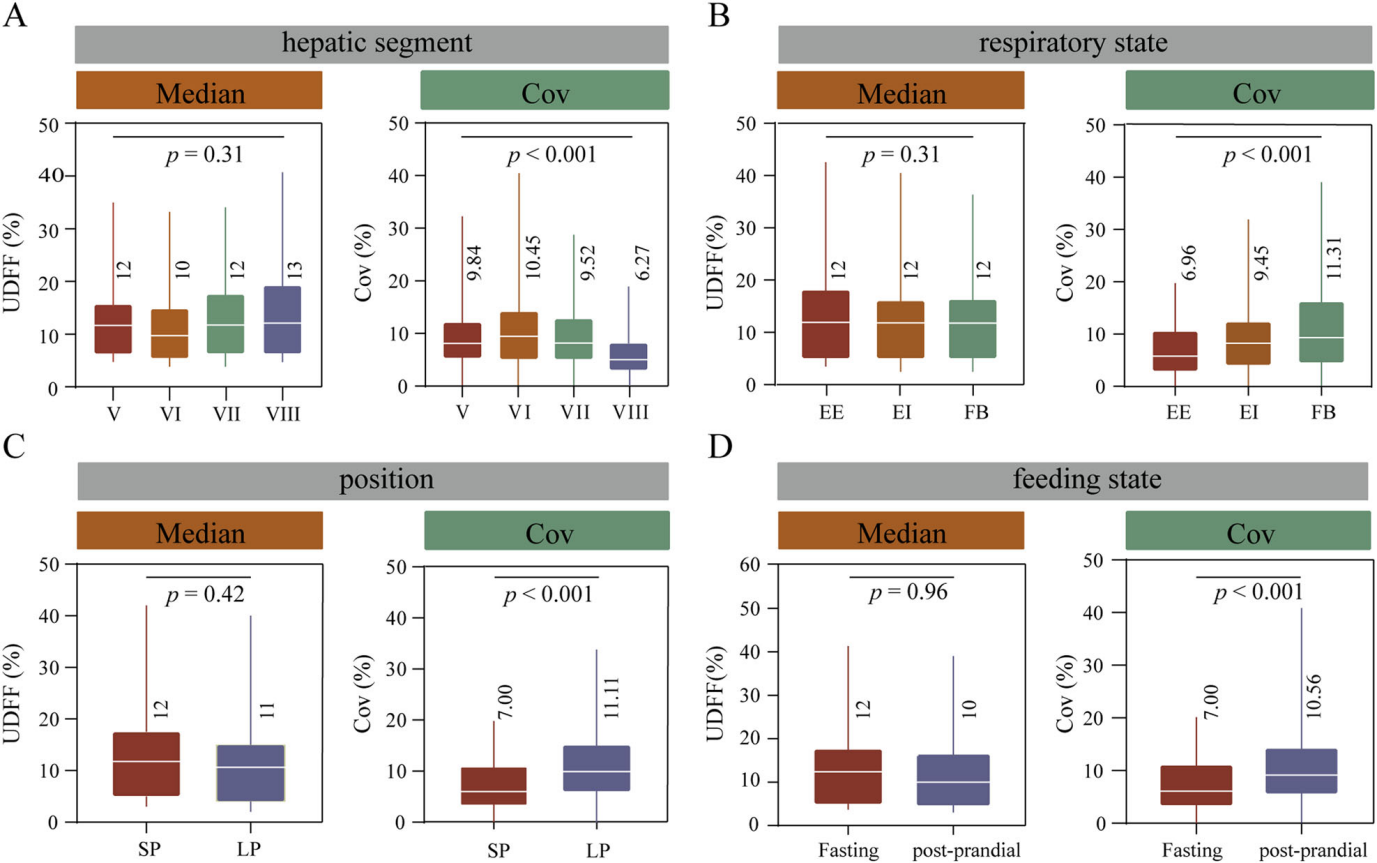
Differences in the UDFF values under various measurement conditions. **A** The variation in different liver segments. **B** The variation in different respiratory states. **C** The variation in different positions. **D** The variation in different feeding states. EE, end of expiratory; EI, end of inspiratory; FB, free breathing; SP, supine position; LP, lateral position. The data did not satisfy a normal distribution and homogeneity of variance

As the UDFF values showed high inter-observer and intra-observer agreement, we further compared the UDFF values in different measurement states. The results are shown in Fig. 3. The median of UDFF values in different hepatic segments were 12 (V), 10 (VI), 12 (VII), and 13 (VIII), respectively; The median UDFF values in different respiratory states were 12 (EE), 12 (EI), and 12 (FB), respectively; The median of UDFF values in different positions were 12 (SP) and 11 (LP). The median UDFF values in different feeding states were 12 (fasting) and 10 (post-prandial). However, none of these differences were statistically significant (*p* > 0.05). On the other hand, the Covs in different measurement states are significantly different (*p* < 0.05). The mean Covs for segments V, VI, VII, and VIII were 9.84%, 10.45%, 9.52%, and 6.27%, respectively; The mean Covs for EE, EI, and FB were 6.96%, 9.45%, and 11.31%, respectively; The mean Covs for SP and LP were 7% and 11.11%; The mean Covs for fasting and post-prandial were 7% and 10.56%. The results showed that the segment VIII, end-expiratory, supine, and fasting groups possessed smaller Covs at different measurement states.

### Correlation of UDFF values with the hepatic steatosis grades and HRI

**Fig. 4:**
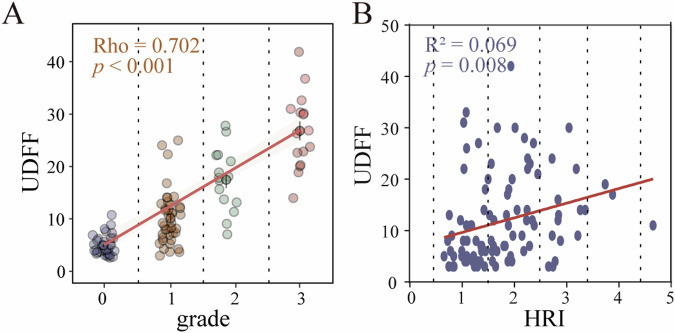
Scatter plots show the correlation of the UDFF values with the hepatic steatosis grades and HRI. **A** Correlation between hepatic steatosis grades and UDFF. **B** Correlation between HRI and UDFF

The UDFF values and hepatic steatosis grades had a significant correlation by a correlated statistical analysis of Spearman’s Rho. The rank correlation coefficient is 0.702 (*p* < 0.05) (Fig. 4A). The Person correlation analysis showed that UDFF had a positive correlation with HRI values (*R*^2^ = 0.069, *p* < 0.05) (Fig. 4B).

### Linear regression analysis of factors affecting the UDFF and auto-pSWE values

**Fig. 5:**
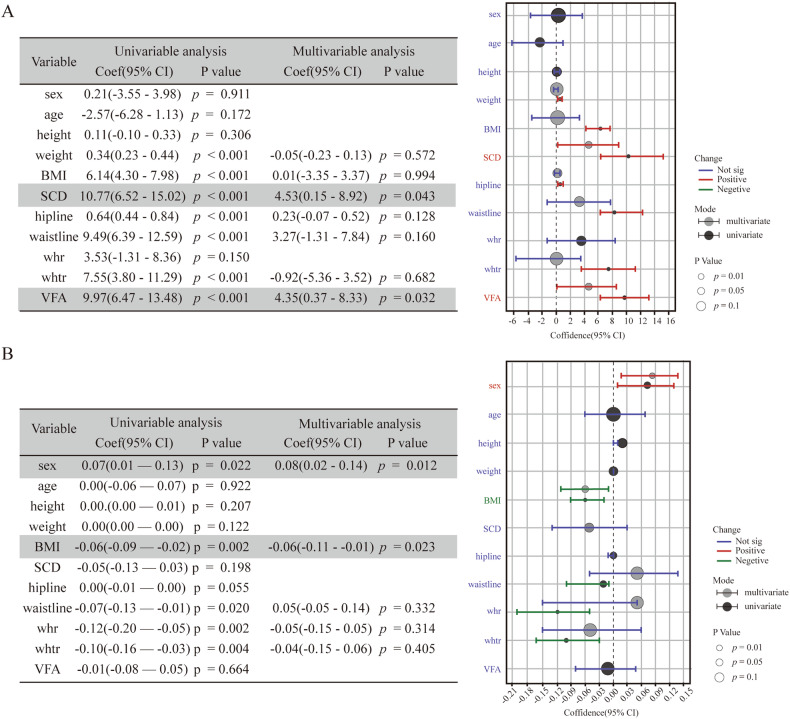
The linear regression analysis of measurement parameters. **A** The liner regression analysis of UDFF. **B** The liner regression analysis of auto-pSWE. BMI, body mass index; SCD, skin-liver capsule distance; whr, waist-hip ratio; whtr, waist-height ratio; VFA, visceral fat area

The factors affecting the UDFF and auto-pSWE values are demonstrated in Fig. 5. We included factors in the multivariate linear regression analysis that were significantly less than 0.05 in the univariate regression analysis. According to the univariate analysis, weight (Coef = 0.34, *p* < 0.001), body mass index (BMI) (Coef = 6.14, *p* < 0.001), SCD (Coef = 10.77, *p* < 0.001), hipline (Coef = 0.64, *p* < 0.001), waistline (Coef = 9.49, *p* < 0.001), waist–height ratio (whtr) (Coef = 7.55, *p* < 0.001), and visceral fat area (VFA) (Coef = 9.97, *p* < 0.001) were associated with the UDFF value (Fig. 5A). SCD (Coef = 4.53, *p* = 0.043) and VFA (Coef = 4.35, *p* = 0.032) were factors significantly associated with the UDFF value according to the multivariate linear regression analysis (Fig. 5A). For auto-pSWE, the simple linear regression analysis showed a significant association between it and sex (Coef = 0.07, *p* = 0.02), BMI (Coef = −0.06, *p* = 0.002), waistline (Coef = −0.07, *p* = 0.02), waist–hip ratio (whr) (Coef = −0.12, *p* = 0.002), and whtr (Coef = −0.1, *p* = 0.004) (Fig. 5B). The multiple linear regression showed that the significant predictors were sex (Coef = 0.08, *p* = 0.01) and BMI (Coef = −0.06, *p* = 0.02) (Fig. 5B). The linear multivariate regression analysis indicated a significant positive association between SCD, VFA, and UDFF (*p* < 0.05), on the other hand, sex and BMI were correlated to auto-pSWE, but the correlation is not strong.

### Consistency between US-UDFF and MRI-PDFF

**Fig. 6:**
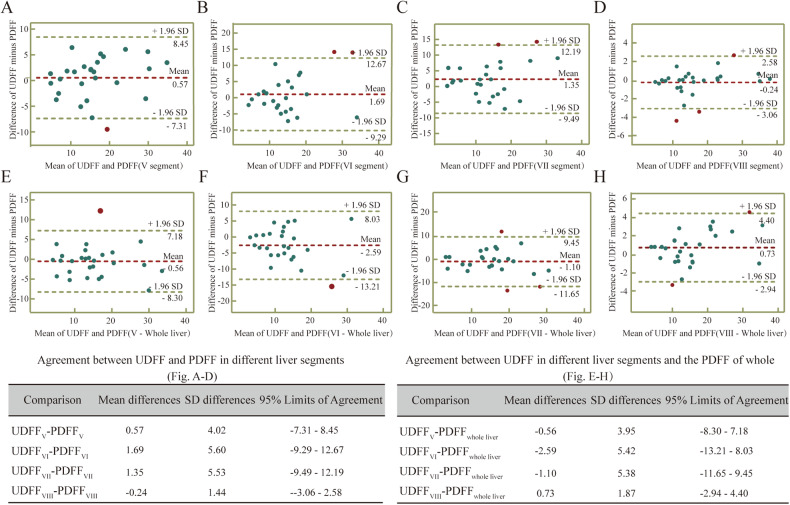
Bland–Altman plot of the difference between UDFF and MRI-PDFF versus the average of the measures. Solid lines demonstrate the mean difference. The dotted lines represent 95% LOA. **A** The difference between UDFF and PDFF for S5. **B** The difference between UDFF and PDFF for S6. **C** The difference between UDFF and PDFF for S7. **D** The difference between UDFF and PDFF for S8. **E** The difference between UDFF of S5 and PDFF of whole liver. **F** The difference between UDFF of S6 and PDFF of whole liver. **G** The difference between UDFF of S7 and PDFF of whole liver. **H** The difference between UDFF of S8 and PDFF of whole liver

We measured MRI-PDFF in 28 of 105 subjects. Of these, 25 were from the SLD group and 3 were from the non-SLD group. Bland–Altman difference plots were used to assess mean bias and 95% limits of agreement (LOA) between UDFF and MRI PDFF measurements in different liver segments. The mean difference between US-UDFF and MRI-PDFF was small (< 2.59) in the four hepatic segments, as well as between them and the whole liver, and the corresponding 95% limits of concordance were less than 21.96% (Fig. 6). The Bland–Altman analysis showed that the mean differences in prediction errors between UDFF and PDFF for liver segments V, VI, VII, VIII were 0.57, 1.69, 1.35, and −0.24, respectively. The result demonstrated that hepatic segment VIII had the lowest mean difference bias compared to other segments. In addition, the concordance analyses showed that the mean difference in prediction error between UDFF for liver segments V, VI, VII, and VIII and PDFF for the whole liver was −0.56, −2.59, −1.10, and 0.73, respectively. The above results suggested both segments V and VIII possessed lower mean difference bias, but segment VIII had smaller concordance boundaries, which may imply that it possesses more stable concordance.

## Discussion

B-mode ultrasound is the imaging technique most commonly used to assess fatty liver. Our study found a positive correlation between UDFF and pre-existing parameters of hepatic steatosis (visual grading and HRI) (*p* < 0.01). This suggests that UDFF is promising in the diagnosis of fatty liver. It is necessary to assess the repeatability and reproducibility of ultrasound technology before further application. Our study confirms the good to excellent reliability of UDFF and auto-pSWE in measuring the adult liver (intra-observer: ICC_UDFF_ = 0.96, ICC_auto-pSWE_ = 0.82; inter-observer: ICC_UDFF_ = 0.94, ICC_auto-pSWE_ = 0.80). The experience and technical proficiency of the doctors may affect the results [14]. The phased measurements in this study showed that training improved operator stability.

Given the excellent performance of UDFF in consistency, we investigated its variability under different measurement states to further explore its stability. Firstly: liver segments. Regional differences in portal perfusion may contribute to spatial heterogeneity of liver fat infiltration [15, 16]. Studies have confirmed that in the right liver, S8 has the highest fat content [15], whereas S5, S7, and S8 have less variability in measurements [17]. Our study did not find spatial variability in fat distribution within the right liver (*p* > 0.05), while a statistically significant difference was found in the mean variability of UDFF. Namely, S8 had the lowest mean variability (Cov = 6.27%), and the highest efficiency rate (100%), whereas S6 had the highest mean variability (Cov = 10.45%) and the lowest efficiency rate (84.76%). Secondly: respiratory state. Breathing motion causes image jitter, so most liver imaging techniques are performed with breath-holding [18], which is currently the recommended measurement state by UDFF manufacturers. It has been demonstrated that respiration may affect intrahepatic blood volume and thus alter liver stiffness [19]. However, it has been shown that respiration does not appear to affect the measurement of steatosis [20–22]. Although no differences in fat content between respiratory states were observed in this study either, the more minor changes in UDFF measurements were found under end-expiration (Cov = 6.96%). Thirdly, body position. The acoustic shadow of the rib in left lateral recumbency causes instability of the acoustic window thereby increasing the Cov of the measurement [23]. Similar results were found in our study. The variability of UDFF values was significantly higher in the left lateral recumbent (Cov = 11.11%) than in the SP (Cov = 7.00%). Fourthly: Ingestion. The physiological increase in intrahepatic blood flow induced by ingestion may affect the measurement of hepatic steatosis and elasticity [24]. Ratchatasettakul et al showed that ingestion can lead to a decrease in steatosis measurements [25]. In this study, the UDFF measurements were repeated 2 h after the meal and showed no significant increase or decrease in UDFF values, but a significant decrease in stability.

This study also analyzed the influences associated with the parameters. The single-factor linear regression analysis revealed that UDFF was correlated with weight, BMI, SCD (skin–liver capsule distance), waistline, hipline, whtr, and VFA, which is consistent with previous studies. Whereas, Multiple analyses showed that VFA and SCD were independent factors that significantly influenced UDFF values. VFA and SCD reflect visceral and subcutaneous fat accumulation and distribution, respectively [26, 27]. Recent studies have shown that VFA and SCD are more strongly correlated with hepatic steatosis than traditional obesity metrics such as BMI [28], which was also confirmed in our study. In addition, our findings suggest that gender and BMI are independent factors influencing auto-pSWE values, but the regression coefficients indicate that this effect is small. To further analyze the accuracy of UDFF in diagnosing hepatic steatosis, we discussed the concordance between UDFF and PDFF by plotting Bland–Altman plots. The results showed that S8 had the highest concordance between UDFF and PDFF. In addition, the UDFF values of S5 and S8 agreed with whole liver PDFF. The reproducibility of quantitative ultrasound parameters is critical for the clinical management of patients with SLD. Both AC and BSC are important parameters for quantifying hepatic steatosis, previously studies have explored their repeatability. It has been reported that the inter-observer ICCs for AC and log-transformed BSC were 0.86–0.88 and 0.87–0.88, respectively [29]. They also found that the inter-platform ICC was 0.77 for AC and 0.70 for log-transformed BSC [10]. Our results indicated that the UDFF calculated from these two parameters had good reproducibility, with both inter- and intra-observer ICC greater than 0.9. However, our study also suggested that the influence of factors such as respiratory status, liver segments, body positions, and feeding status on the measurement stability still needs to be noted during actual clinical application. In addition, the experience of observers and strict adherence to the manufacturer’s standardized protocols are also important.

Our study also has limitations. Firstly, the sample size was limited. More large clinical cohorts are still needed to validate our results. In addition, we only performed MRI-PDFF in 28 subjects, which may affect the universality of the results of consistency between UDFF and PDFF. Secondly, since auto-pSWE is a further refinement of the well-established pSWE technique, we did not analyze differences in auto-pSWE values for different measurement states. Thirdly, we did not use MRI-MRE to assess participants’ stiffness and could not assess the consistency between auto-pSWE and the quantitative detection technology.

## Conclusion

UDFF and auto-pSWE had good repeatability and reproducibility in assessing the adult liver. Furthermore, we suggest that S8, end-expiration, fasting, and supine might be optional when considering the measurement variability.

## Abbreviations

AC: Attenuation coefficient
ALD: Alcohol-related liver disease
BMI: Body mass index
BSC: Backscatter coefficient
Cov: Coefficient of variation
HRI: Hepatic renal index
ICC: Intraclass correlation coefficient
LOA: Limits of agreement
MASLD: Metabolic dysfunction-associated steatotic liver disease
PDFF: Proton density fat fraction
SCD: Skin–liver capsule distance
SLD: Steatotic liver disease
UDFF: Ultrasound-derived fat fraction
VFA: Visceral fat area
